# Barriers to and Facilitators of Implementing Team-Based Extracorporeal Membrane Oxygenation Simulation Study: Exploratory Analysis

**DOI:** 10.2196/57424

**Published:** 2025-01-24

**Authors:** Joan Brown, Sophia De-Oliveira, Christopher Mitchell, Rachel Carmen Cesar, Li Ding, Melissa Fix, Daniel Stemen, Krisda Yacharn, Se Fum Wong, Anahat Dhillon

**Affiliations:** 1 Department of Surgery, Keck School of Medicine, University of South California, Los Angeles, CA, United States; 2 Office of Performance & Transformation, Keck Hospital of USC, Los Angeles, CA, United States, United States; 3 Department of Nursing, Keck Hospital of USC, Los Angeles, CA, United States, United States; 4 Department of Population and Public Health Sciences, Keck School of Medicine, University of Southern California, Los Angeles, CA, United States, United States; 5 Deparment of Respiratory Therapy, Keck Hospital of USC, Los Angeles, CA, United States, United States; 6 Department of Anesthesia, Kaiser Permanante, Los Angeles, CA, United States; 7 Department of Anesthesia Critical Care Medicine, Keck School of Medicine, University of South California, Los Angeles, CA, United States, United States

**Keywords:** intensive care unit, ICU, teamwork in the ICU, team dynamics, collaboration, interprofessional collaboration, simulation, simulation training, ECMO, extracorporeal membrane oxygenation, life support, cardiorespiratory dysfunction, cardiorespiratory, cardiology, respiratory, heart, lungs

## Abstract

**Introduction:**

Extracorporeal membrane oxygenation (ECMO) is a critical tool in the care of severe cardiorespiratory dysfunction. Simulation training for ECMO has become standard practice. Therefore, Keck Medicine of the University of California (USC) holds simulation-training sessions to reinforce and improve providers knowledge.

**Objective:**

This study aimed to understand the impact of simulation training approaches on interprofessional collaboration. We believed simulation-based ECMO training would improve interprofessional collaboration through increased communication and enhance teamwork.

**Methods:**

This was a single-center, mixed methods study of the Cardiac and Vascular Institute Intensive Care Unit at Keck Medicine of USC conducted from September 2021 to April 2023. Simulation training was offered for 1 hour monthly to the clinical team focused on the collaboration and decision-making needed to evaluate the initiation of ECMO therapy. Electronic surveys were distributed before, after, and 3 months post training. The survey evaluated teamwork and the effectiveness of training, and focus groups were held to understand social environment factors. Additionally, trainee and peer evaluation focus groups were held to understand socioenvironmental factors.

**Results:**

In total, 37 trainees attended the training simulation from August 2021 to August 2022. Using 27 records for exploratory factor analysis, the standardized Cronbach α was 0.717. The survey results descriptively demonstrated a positive shift in teamwork ability. Qualitative themes identified improved confidence and decision-making.

**Conclusions:**

The study design was flawed, indicating improvement opportunities for future research on simulation training in the clinical setting. The paper outlines what to avoid when designing and implementing studies that assess an educational intervention in a complex clinical setting. The hypothesis deserves further exploration and is supported by the results of this study.

## Introduction

Simulation training for extracorporeal membrane oxygenation (ECMO) has become standard practice for reinforcing technical skills, facilitating troubleshooting, and building teamwork [1]. ECMO is a critical tool in the care of severe cardiorespiratory dysfunction among patients of all ages [1]. Within the intensive care unit (ICU), ECMO is one of the most complicated therapies, requiring not only extensive knowledge of cardiopulmonary physiology and expertise with intricate circuit components but also skills to rapidly respond to emergent situations [2]. Therefore, high-fidelity simulation trainings are critical to practice skills and work through different emergency scenarios, such as the blood pump falling from the drive unit [3]. A randomized control study concluded that exposure to high-fidelity simulated ECMO emergencies leads to significant improvements in technical and behavioral skills among clinicians. This study demonstrated that simulation training creates a learning environment that replicates the clinical setting and fosters acquisition of cognitive, technical, and behavioral skills [4].

The Extracorporeal Life Support Organization, an international nonprofit association of health care institutions focused on ECMO research and education, recommends simulation training didactic sessions, water drills, animal sessions, and bedside training [5]. However, a randomized controlled trial published in *Critical Care Medicine* compared traditional water drill with simulation and found that simulation-based training is more effective than traditional training [6]. Water-based drills do not offer the same hands-on experience of real-time troubleshooting, and the use of animals is expensive and complex [6]. Nevertheless, traditional and simulation-based training are both beneficial to ECMO education. The benefits of simulation training on reinforcing skills have been noted in the literature [3,7-9]. Therefore, Keck Medicine of the University of California (USC) has held ECMO simulation-training sessions since 2013 for nursing education and 2021 for interprofessional simulation to reinforce and improve providers knowledge and hands-on skills in high-risk, low-frequency scenarios at no risk to patients [6].

We implemented simulation-based ECMO training to improve interprofessional collaboration through increased communication and enhanced teamwork. Moreover, the intention of the simulation training was to strengthen collaboration skills and increase confidence in providers to work through emergency scenarios. The specific aim of the study was to understand the impact of our simulation training approach on interprofessional collaboration. However, ICU staffing models impacted the ability to execute the study design as intended. This paper outlines the original study design, the challenges the research team faced during the study, and the lessons learned to ensure future studies mitigate the challenges posed by real-world ICU operations. Our primary outcome shifted to the development and validation of measurement tools and offers recommendations for the evaluation of simulation approaches in future studies.

## Methods

### Overview

**Figure 1.**
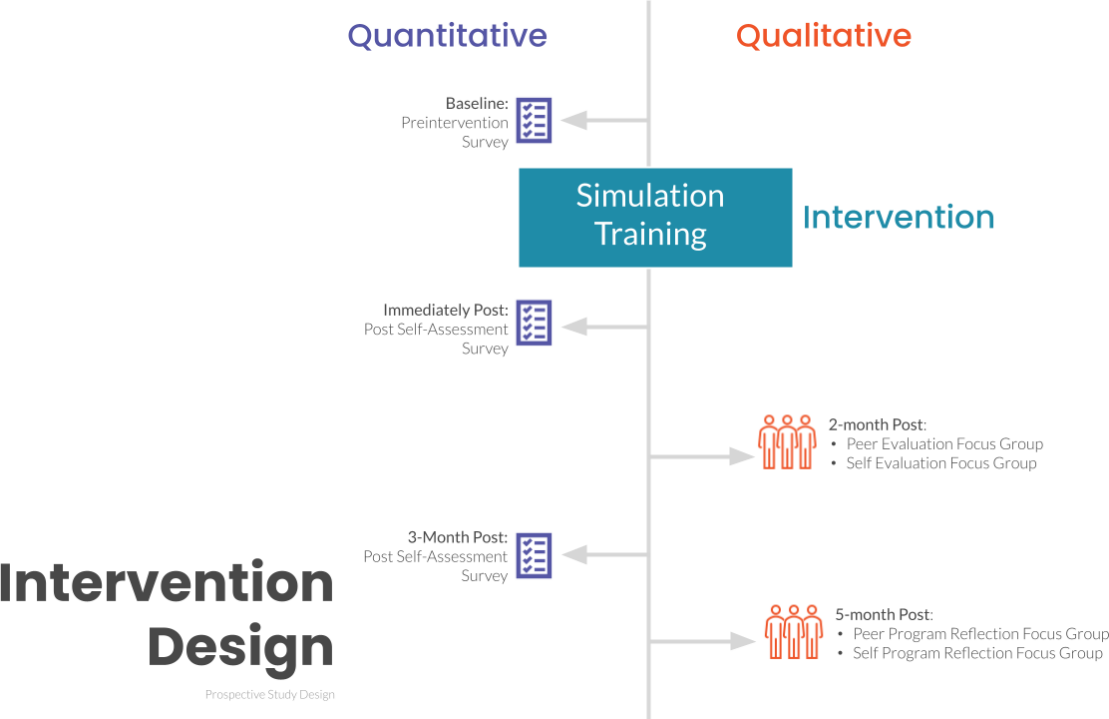
Study design.

This was a single-center, mixed methods study of the Cardiac and Vascular Institute (CVI) ICU at Keck Medicine of USC conducted from September 2021 to April 2023. The study was designed to elicit quantitative feedback through an electronic survey before and post a voluntary training simulation exercise, and qualitative feedback from a participants via a series of focus groups that incorporated self- and peer evaluations (Figure 1).

### Participants

A census sampling strategy was used to recruit participants; in other words, all trainees that participated in the training were offered participation in the study. Participants included: (1) trainee physician fellows and residents in the CVI ICU who were offered attendance to the simulation training by their program director, and (2) peer evaluators, including the CVI ICU’s Medical Director, nurse manager, nurse clinical educator, and lead respiratory therapist. Participant trainees attended a single 1-hour simulation training with roles played by clinical staff members from the CVI ICU, including an intensivist, clinical nurse educator, and respiratory therapist. To be eligible for the study, participants needed to be in a fellowship on rotation at Keck Medicine of USC. Fellows were recruited by intensivist leaders of the CVI from departments that rotated through or interacted with the CVI ICU (Pulmonary Critical Care Medicine, Cardiology, Anesthesia, Surgical Critical Care, and Cardiac Surgery). All study recruitment took place via email by the CVI Medical Director and Program Director to physicians in fellowship based on department and rotation schedule from pulmonary critical care medicine, surgical critical care medicine, cardiac surgery, and cardiology.

### Simulation Training

Simulation training was designed as part of the continuing clinical education offered to the clinical team for 1-hour monthly, where participants attended a single session. Simulations were designed to focus on the interprofessional collaboration and decision-making needed to evaluate a patient for the initiation of ECMO therapy (Table S1 in Multimedia Appendix 1). Initially, low-fidelity simulations were held in a conference room using (1) a resuscitation training mannequin, (2) simulated vital signs via a hospital patient monitor connected to a rhythm simulator, (3) simulated intravenous access, (4) simulated medications, and (5) emergency equipment. In January 2022, collaboration with the Keck School of Medicine Simulation department allowed for training to be held in a simulation lab with a high-fidelity simulation mannequin and integrated simulation software LLEAP, version 8.5 from Laerdal. The availability of a higher fidelity training environment was meant to improve the training experience of the learners.

Each training session began with an orientation to the simulation environment and assigned roles. The scenario (Table S1 in Multimedia Appendix 1) was created to include relative contraindications to ECMO therapy and a potentially reversible condition that led to a cardiac arrest requiring resuscitation. Participants were assigned into roles of primary physician, code blue response provider, and cardiac surgeon prior to entering the simulation and entered the scenario when prompted by the facilitator or requested during the simulation by another participant. The patient was introduced to the learners as a 65-year-old female in-patient on a hospital cardiac telemetry unit with a past medical history of coronary artery disease, congestive heart failure, and peripheral vascular disease. The simulation began when a facilitator in the role of the patient’s nurse requested help from a participant. The simulated patient was initially responsive with complaints of palpitations and shortness of breath with intermittent ventricular tachycardia displayed on the cardiac monitor. The simulated patient then became unresponsive in persistent ventricular tachycardia, and the imbedded facilitator activated the resuscitation team. When the simulated patient’s cardiac rhythm changes, the participants performed the roles of a code blue response, including coordinating the resuscitation, performing a simulated echocardiograph, and performing simulated invasive procedures including endotracheal intubation, arterial line insertion, and central line insertion. The participants collaborated to identify the candidacy of the simulated patient for ECMO therapy and proceeded to participate in a moderate-fidelity mock cannulation with ECMO training equipment. The participant in the surgeon role chose a method and site of cannulation for the simulated patient, and a practice ECMO circuit was connected to the simulator. Participants proceeded to respond and troubleshoot as the patient was set to be initially unstable during the transition to ECMO support. The simulated patient remained in ventricular tachycardia, and the participants were required to decide whether to continue attempting interventions, including, for example, chest compressions, medication, and defibrillation once the patient was placed on ECMO. The simulation ended when the patient was stabilized on ECMO and the participants decided to transfer the patient to the ICU. Areas of safety concern (Figure S1 in Multimedia Appendix 2) were emphasized in the training as points for communication to consider the decision to initiate ECMO with an unstable patient. A postsimulation debriefing session was facilitated by the simulation faculty.

### Qualitative Approach

To understand the social environment factors the simulation training impacted, a total of 12 qualitative focus groups were planned (Figure 1). The 12 interviews were divided into 6 focus groups with the trainee attendees of the simulation training as a self-evaluation and 6 focus groups with colleague participants as a peer evaluation (Figure S2 in Multimedia Appendix 3). Each focus group was designed to have 2‐4 participants. The study was designed to use the same peer evaluators for each peer evaluation focus group for the study duration. Each peer evaluation focus group was meant to target the evaluation of the individuals in the 6 simulation cohorts with a total of 4 peer participants. The focus groups were designed to be a duration of 30 minutes. Questions were developed to assess how the simulation training impacted the trainees’ practice related to collaboration and teamwork (Table S3 in Multimedia Appendix 4). Questions were reviewed by the study expert in mixed methods study application.

Interviews were conducted by the simulation facilitators experienced in ECMO therapy and simulation education. Sessions were recorded using the Voice Memo application (Apple, Inc.). Once the focus groups were completed, the simulation facilitators sent the audio file to the data management author for transcription. The transcribed focus group sessions were de-identified, then uploaded and stored to a HIPAA (Health Insurance Portability and Accountability Act)-compliant Microsoft OneDrive. All audio and video files containing identifiers were deleted following transcription. Transcription documents were reviewed and coded for key themes using grounded theory methodology, an iterative process that will identify conceptual categories emerging from the comparative analyses of the data.

### Quantitative Approach

An electronic survey was distributed with a QR code in person and electronically via email using Qualtrics XM software version December 2019. A total of 49 questions were posed to trainees across the pretraining, posttraining, and 3-month posttraining questionnaires (Table S2 in Multimedia Appendix 5). Teamwork-focused questions were obtained from the validated Mayo High Performance Teamwork scale (16 questions) [10]. The remaining questions regarding the effectiveness of training were devised using the Kirkpatrick Training Evaluation Framework as a basis for query design [11]. The study biostatistician performed a psychometric review to assess the validity and reliability of the survey questions. The use of existing validated tools ensured high reliability and validity of the teamwork elements of the survey tool [10]. Additionally, field tests (1 MD, 2 RNs, and 1 RT) of the survey tool showed an average survey duration of 7 minutes or less and promoted consistent comprehension of the study questions across individuals.

Exploratory factor analysis was conducted to assess questions reflecting underlying factors. The number of factors included in the final model was determined by eigenvalue and Scree plot. In the final factor pattern table, questions with a value>0.4 were considered well loaded for the factor. The Validated Mayo High Performance Teamwork scale (16 questions) was used as a sum score as recommended by the study [10]. Secondary outcomes analysis included department-level patient mortality, average device days on ECMO, decannulation percentage, and percentage of staff that had simulation training.

### Ethical Considerations

The study was approved by the University of Southern California institutional review board (UP-21-01021). Prior to participation, all study participants were required to sign an informed consent form, thereby confirming their voluntary engagement in the survey process. The study data were anonymous.

## Results

**Figure 2.**
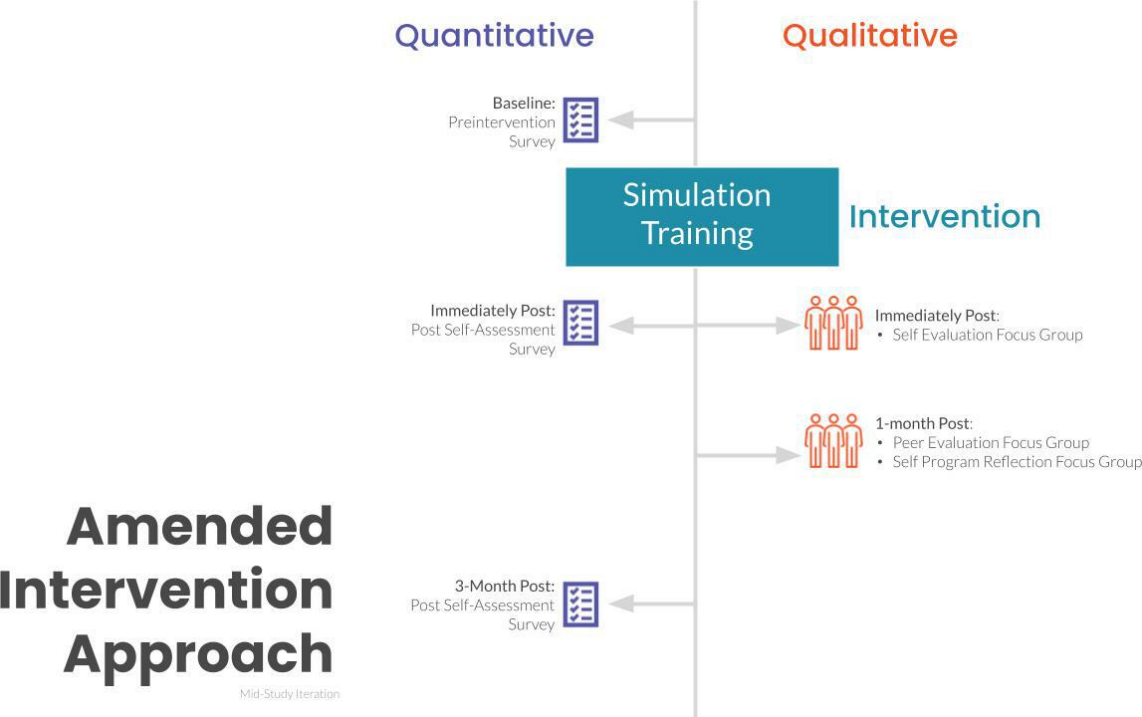
Amended study design.

A total of 37 trainees attended the training simulation from August 2021 to August 2022. However, only 7 trainees opted to participate in the qualitative portion of the study. Due to lack of participant engagement, mid-study the study design was amended to increase study participation (Figure 2). The quantitative approach remained as originally designed; however, the analysis approach was done descriptively due to the inability to compare pre- and postsurvey results on an individual basis. In other words, survey analysis aggregated all preresponses and then postresponses to compare the pregroup and postgroup responses. The qualitative approach shifted to trainee participants attending a total of 2 focus groups, an initial self-evaluation immediately following simulation and a 1-month post-program reflection if they were available (Figure S3 in Multimedia Appendix 6).

### Qualitative Approach

A total of 4 focus groups were conducted between January 2022 and August 2022 including 2 trainee self-evaluations (n=7) immediately post the training simulation and 2 peer evaluations 2-months post-training evaluation with the group of 4 peers. Focus groups for participants between August 2021 and December 2021 were not coordinated due to lack of trainee engagement in study participation. The 2 peer focus groups held highlighted an issue in trainee exposure with the peer team. Peer participants noted that they had limited clinical working exposure to the trainees being evaluated due to the nature of the fellow’s rotation in the ICU. Meaning, peers did not have any recollection of working with the trainees prior to and following the simulation training to provide an appropriate evaluation of their skills in teamwork during ECMO therapy decision-making. In other words, peers remembered a trainee prior to or following the simulation, but not both. The 2 trainee self-evaluations that occurred highlighted themes that showed the simulation training benefited the trainees, including that the simulation training resulted in (1) working together as a stronger and more confident team because of simulation and (2) the creation of a space to improve communications, decision-making, and express concerns (Table S5 in Multimedia Appendix 7).

### Quantitative Approach

All trainees were asked to complete the pre-, immediately post-, and 3-month surveys as part of the simulation training experience. There were a total of 37 entries recorded for the pre-survey, yielding a 100% response rate. Of those entries, 9 records were excluded due to lack of record ID or mismatching question numbers, leaving 28 entries for analysis. There was a total of 35 entries recorded for the postsurvey, yielding a 95% response rate. Following data cleaning, 9 records were excluded, leaving 26 postsurvey entries for analysis. There were a total of 2 entries for the 3-month posttraining survey, yielding a 5.4% response rate. The 3-month postsurvey results were excluded from analysis due to the low response rate. Table S4 in Multimedia Appendix 8 details the survey results of each question.

**Figure 3.**
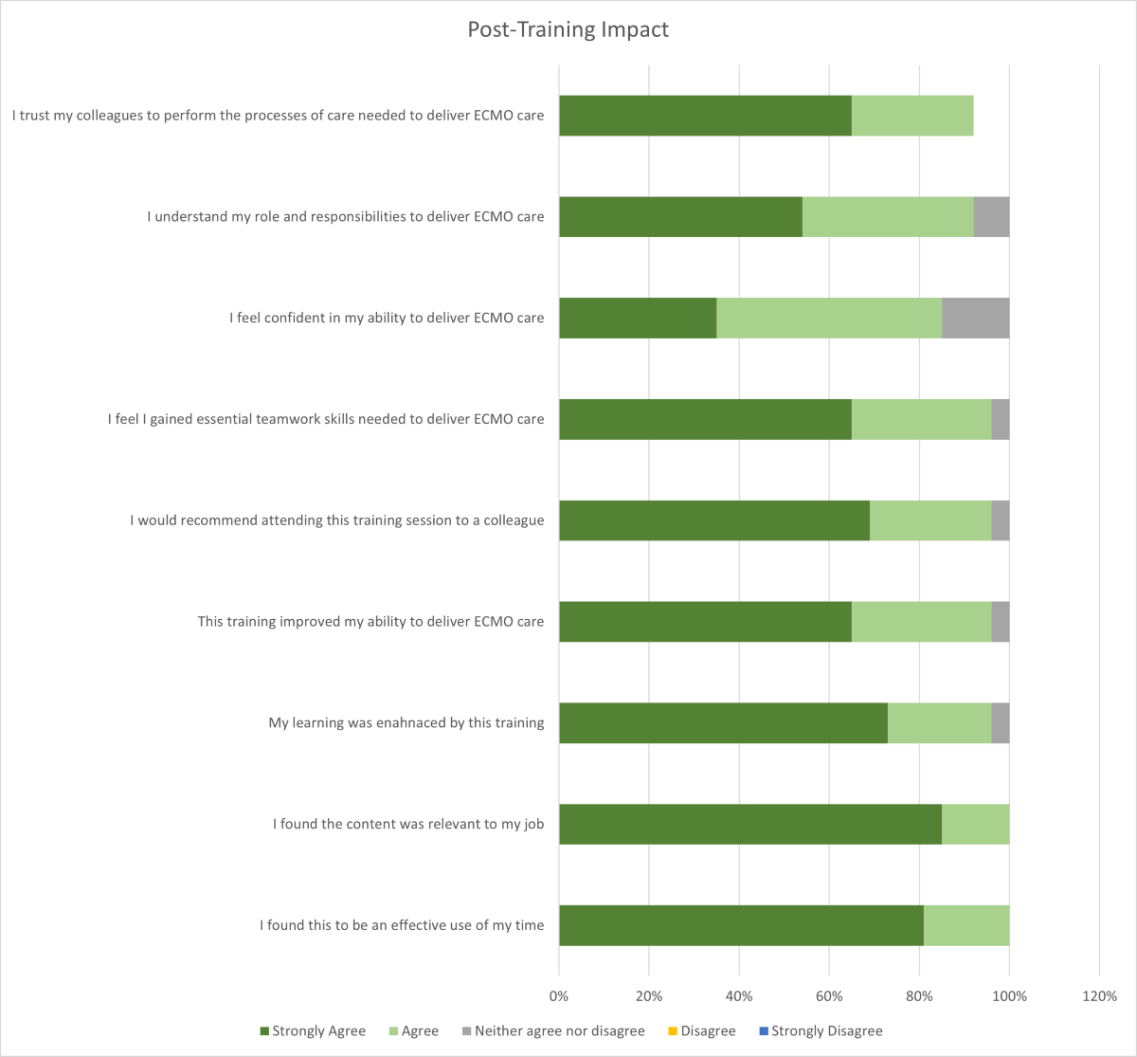
Posttraining impact. ECMO: extracorporeal membrane oxygenation.

Questions posed in the post-survey focused on assessing levels 1 (reaction) and 2 (learning) of the Kirkpatrick Training Evaluation Framework demonstrated a high level of agreement for positive postsimulation training impact (Figure 3). Additionally, an increase in knowledge and understanding were noted descriptively when comparing the pre- and postresponses of the survey (Table S4 in Multimedia Appendix 8). For example, question “I understand the mechanism for activating ECMO at Keck Hospital” in the presurvey 46% of respondents agreed or strongly agreed to the statement compared to the postsimulation training survey where 100% of participants responded with a level of agreement. Levels 3 (impact) and 4 (results) of Kirkpatrick’s framework were unable to be assessed due to the low response rate for the 3-month postsimulation survey.

The Mayo Teamwork Scale was used to understand changes in the trainee’s perspective on teamwork before and after the simulation training. The 16 focused teamwork questions demonstrated a positive shift in teamwork ability; that is, in the presurvey participants had a 71% average response on performing each question consistently, postsurvey showed an increase of the average to 95% consistently.

### Exploratory Factor Analysis

An exploratory factor analysis was conducted to validate the use of the survey in evaluating the effectiveness of team training in ECMO simulation (Table 1). Entries with missing questions were excluded from the factor analysis; that is, only records with all questions answered were used. A total of 28 data points were available in the presurvey and 26 for the postsurvey. For the factor analysis, 0.4 was used as the cutoff. For the Mayo High-Performance Teamwork scale, 23 records were collected with a mean sum score of 26.87 (SD 6.41) at the presurvey and 21 records at the postsurvey with a mean sum score of 31.1 (SD 1.88). For the presurvey, we asked six 5-likelihood questions. Using 27 records for exploratory factor analysis, only 1 question, “Q5,” did not reflect the underlying factor. Standardized Cronbach α was 0.686 when using all 6 questions. After excluding Q5, the standardized Cronbach α is 0.717. For the postsurvey, 26 records were used for analysis with 3 factors. Standardized Cronbach α was 0.919 when using all 15 questions. For questions from the Mayo High-Performance Teamwork scale, 21 records were used for analysis with a mean sum score of 31.1 and a SD of 1.88.

**Table 1.** Exploratory factor analysis.

		Factor 1	Factor 2	Factor 3
**Presurvey**				
	1. Please rate the following statements—I understand the mechanism for activating ECMO^a^ at Keck Hospital	0.556	—^b^	—
	2. Please rate the following statements—I understand my role in a bedside cannulation	0.755	—	—
	3. Please rate the following statements—I feel comfortable using the ECMO equipment specific to my role	0.723	—	—
	4. Please rate the following statements—I feel comfortable using the 2-challenge rule	0.478	—	—
	5. Please rate the following statements—I feel confident to voice concerns to leadership during a critical situation	0.201	—	—
	6. Please rate the following statements—I trust my colleagues to perform the processes of care needed to deliver ECMO care	0.417	—	—
**Postsurvey**				
	1. Please rate the following statements—I understand the mechanism for activating ECMO at Keck Hospital	—	0.880	—
	2. Please rate the following statements—I understand my role in a bedside cannulation	—	0.602	—
	3. Please rate the following statements—I feel comfortable using the ECMO equipment specific to my role	—	—	0.412
	4. Please rate the following statements—The initiating team communicates efficiently during a bedside cannulation	0.543	0.456	—
	5. Please rate the following statements—I feel comfortable using the 2-challenge rule	—	—	0.659
	6. Please rate the following statements—I feel confident to voice concerns to leadership during a critical situation	—	0.705	—
	7. Please rate the following statements—I found this to be an effective use of my time	0.851	—	—
	8. Please rate the following statements—I found the content was relevant to my job	0.813	—	—
	9. Please rate the following statements—My learning was enhanced by this training	0.927	—	—
	10. Please rate the following statements—This training improved my ability to deliver ECMO care	0.891	—	—
	11. Please rate the following statements—I would recommend attending this training session to a colleague	0.844	—	—
	12. Please rate the following statements—I feel I gained essential teamwork skills needed to deliver ECMO care	0.916	—	—
	13. Please rate the following statements—I feel confident in my ability to deliver ECMO care	0.532	—	0.670
	14. Please rate the following statements—I understand my role and responsibilities to deliver ECMO care	0.695	—	—
	15. Please rate the following statements—I trust my colleagues to perform the processes of care needed to deliver ECMO care	0.788	—	—

a ECMO: extracorporeal membrane oxygenation.

b Not applicable.

### Triangulation of Quantitative and Qualitative Results

Applying procedures of convergent mixed methods design, we converged the quantitative and qualitative results that were obtained separately to obtain a nuanced understanding of the core research aims. The themes identified of teamwork and improved communication in the qualitative analysis were supported by the quantitative survey results (Tables2  3). Qualitative subthemes were supported by the positive shift observed descriptively from the pre- compared to the post-simulation training survey results.

**Table 2.** Triangulation of quantitative and qualitative results between frequently endorsed survey items and themes emerging from postsimulation focus groups (part ).

Question		Agree level (Strongly Agree + Agree), %	Neither agree nor disagree, %	Disagree level (Strongly Disagree + Disagree),	Qualitative theme
**I understand the mechanism for activating ECMO**^a^ **at Keck Hospital**					Working together as a stronger and more confident team because of simulation
	Prestimulation	46	18	36	
	Poststimulation	100	0	0	
**I understand my role in a bedside cannulation**					Working together as a stronger and more confident team because of simulation
	Prestimulation	23	48	30	
	Poststimulation	100	0	0	
**I feel comfortable using the 2- challenge rule**					Working together as a stronger and more confident team because of simulation
	Prestimulation	4	29	68	
	Poststimulation	69	15	15	
**I feel confident to voice concerns to leadership during a critical situation**					Working together as a stronger and more confident team because of simulation
	Prestimulation	78	11	11	
	Poststimulation	96	4	0	
I found this to be an effective use of my time (poststimulation)		100	0	0	Creating a space to improve communications, decision-making, and express concerns via simulation
I found the content was relevant to my job (poststimulation)		100	0	0	Creating a space to improve communications, decision-making, and express concerns via simulation
My learning was enhanced by this training (poststimulation)		96	4	0	Creating a space to improve communications, decision-making, and express concerns via simulation
This training improved my ability to deliver ECMO care (poststimulation)		96	4	0	Creating a space to improve communications, decision-making, and express concerns via simulation
I feel I gained essential teamwork skills needed to deliver ECMO care (poststimulation)		96	4	0	Creating a space to improve communications, decision-making, and express concerns via simulation
I feel confident in my ability to deliver ECMO care (poststimulation)		85	15	0	Creating a space to improve communications, decision-making, and express concerns via simulation
I understand my role and responsibilities to deliver ECMO care (poststimulation)		92	8	0	Creating a space to improve communications, decision-making, and express concerns via simulation
I trust my colleagues to perform the processes of care needed to deliver ECMO care (poststimulation)		92	0	0	Working together as a stronger and more confident team because of simulation

a ECMO: extracorporeal membrane oxygenation.

**Table 3.** Triangulation of quantitative and qualitative results triangulation between frequently endorsed survey items and themes emerging from post-simulation focus groups (part 2).

Question		% Never or rarely	% Inconsistently	% Consistently	Qualitative theme
**A leader is clearly recognized by all team members**					Working together as a stronger and more confident team because of simulation
	Prestimulation	0	41	59	
	Poststimulation	0	17	83	
**Each team member demonstrates a clear understanding of his or her role**					Working together as a stronger and more confident team because of simulation
	Prestimulation	0	37	63	
	Poststimulation	0	8	92	
**The team prompts each other to attend to all significant clinical indicators throughout the procedure or intervention**					Working together as a stronger and more confident team because of simulation
	Prestimulation	0	26	74	
	Poststimulation	0	8	92	
**Disagreements or conflicts among team members are addressed without a loss of situation awareness**					Working together as a stronger and more confident team because of simulation
	Prestimulation	0	30	70	
	Poststimulation	0	4	96	
	Poststimulation	0	4	96	
	Poststimulation	0	0	100	

## Discussion

### Principal Findings

This study was designed to evaluate the impact of ECMO therapy simulation training, specifically focused on enhancing teamwork and communication. The study was successful in validating the survey for future use in assessing the effectiveness of ECMO simulation training in improving teamwork and communication. However, while rigorous and well thought out in design, clear flaws were identified that need to be addressed in future attempts to study this type of simulation exercise. We outline the limitations of the study with recommendations for research with the intention to share what to avoid when designing and implementing studies that assess a clinical education approach in a complex clinical setting. We provide a unique validated tool to assess teamwork and collaboration across clinical disciplines during ECMO therapy, where existing evidence assesses the impact of simulation approaches on knowledge.

### Strengths and Limitations

First, the original focus of the study targeted physician fellows and residents from various clinical teams that practice in the CVI unit. An assumption was made in the study design that peer evaluators would have enough interaction with trainees before and after the simulation training to evaluate changes in their behavior. Due to the nature of the rotation of this participant population, the peers were unable to assess any impact. Additionally, the rotation of the trainees contributed to difficulty in follow-up for study participation in both the quantitative and qualitative aspects of the study. Only 2 responses were received for the 3-month postsimulation training survey, and the qualitative study was altered midstudy to garner more participation in study focus groups. The team was unable to obtain commitment from trainees for the 2-month and 5-month planned focus groups and amended the study for a trainee self-evaluation focus group immediately following the simulation training and 1 month post. The study team was unable to coordinate the 1-month post–focus group due to a lack of availability of the fellow and resident trainees. The lack of participation led to the inability to assess levels 3 and 4 of the Kirkpatrick Training Evaluation Framework [11]. Additionally, the lack of participation reduced the validity of the qualitative data obtained in the focus groups. To generalize the qualitative results of the study, the original target of 6 simulation cohorts with a total of 4 peer participants each would be necessary. We suggest future studies alter the study design to broaden study participants to the entire interprofessional team to ensure the target participant enrollment and focus groups are reached. Second, the trainee rotation also did not guarantee exposure of the trainees to ECMO cannulation postsimulation training to practice the technical skills gained from the simulation training. Third, quantitative results demonstrated there is merit to this training simulation approach. Where there were positive shifts from pre- compared to postsimulation training survey results. However, we were unable to calculate statistical significance in pre- and postresponses due to survey collection methods. Survey participation was anonymous and a routine part of the simulation training program. We were unable to align individual pre- and postsurvey responses to apply this statistical strategy or follow up with specific trainees that missed questions. Fourth, although the trainees had a qualitatively and quantitatively favorable response in ECMO initiation following the simulation exercise per survey results, the study did not conclusively demonstrate their ability to actively use that attained knowledge beyond the original simulation date given the lack of actual cannulations and, again, being observed by staff who could claim that teamwork was significantly improved in future interactions. Lastly, the study team anticipated a larger sample of participants, but the recruitment challenges, focus on physician fellows and residents, and staff shortages due to the impact of the COVID-19 pandemic were severe limitations of the study.

Despite these limitations, the quantitative survey results descriptively highlighted the positive impact on the trainees. Level 1 questions of the Kirkpatrick Training evaluation [11] were met with a strong level of agreement, with no level of disagreement responses (Figure 1). Additionally, each of the level 2 questions shifted to a higher level of agreement post the simulation training. The same was true for the responses to the Mayo Teamwork Scale, where each response shifted to more consistent teamwork behavior pre- and postsimulation training (Table S4 in Multimedia Appendix 8).

We know team-based interprofessional care has historically demonstrated gains in positive patient outcomes in the ICU and is seen as the solution to reduce medical errors and poor quality [12-15]. Moreover, a key component of ECMO care is interprofessional collaboration, as it requires a large and multifaceted team of providers collaborating to carry out complementary tasks to one another [16]. Simulation-based team training can cultivate and preserve interprofessional teamwork and communication [16]. However, collaboration across the care team is not a standard topic covered in clinical curriculum [13,15]. We believe our survey results support the merit of our teamwork-focused simulation training approach and its ability to foster a higher level of collaboration when the clinical team is faced with deciding to initiate ECMO therapy in the cardiac and vascular patient population. These findings highlight the importance of simulation training from other innovative ways of ECMO skills training, such as game-based mobile apps, which might not cultivate a teamwork approach to the same extent [17]. This approach could be applied to supplement the lack of practical teamwork focus in today’s clinical curriculum.

Despite the identified limitations, the study underscored several positive aspects of ECMO simulation training. The quantitative survey results notably revealed a significant positive impact on the trainees. Level 1 questions of the Kirkpatrick Training evaluation demonstrated a strong level of agreement without any disagreement responses, indicating a high degree of satisfaction with the training (Figure 1). Furthermore, each of the level 2 questions exhibited a shift towards higher levels of agreement postsimulation training. Similarly, responses to the Mayo Teamwork Scale demonstrated a consistent improvement in teamwork behavior before and after simulation training (Table S4 in Multimedia Appendix 8). This reaffirms the notion that team-based interprofessional care, a cornerstone in ICU settings, can lead to enhanced patient outcomes and reduced medical errors. The study’s focus on cultivating interprofessional collaboration through simulation-based training aligns with the demands of ECMO care, which relies heavily on coordinated efforts among various health care professionals. These findings highlight the effectiveness of the teamwork-focused simulation training approach in preparing clinical teams to make critical decisions regarding ECMO therapy in the cardiac and vascular patient population. Moreover, they emphasize the importance of incorporating such training into clinical curricula to ensure a holistic approach to health care education. The study’s insights pave the way for future research endeavors to further explore and refine the application of simulation training in improving teamwork and patient outcomes in complex clinical settings. By addressing the outlined recommendations and leveraging innovative approaches, such as virtual reality simulation, the medical community can continue to advance ECMO care delivery and interprofessional collaboration, ultimately enhancing patient care outcomes.

Future research may build upon the learning of this study to strengthen the understanding of a teamwork-focused simulation approach. We would encourage implementation of the following and plan for our future studies to include (1) continuing the study with the entire interprofessional team, using the survey to build on exploratory factor analysis that validated the survey questions and provide a confirmatory factor analysis to validate results; (2) emphasize established continuity with the learners and the peer evaluators in the study design to mitigate the limited interactions with the study participants outside of the actual simulation and during their clinical rotation; (3) training operational staff participating in gathering data on best practices of data collection for operations and research; (4) the team would encourage incorporating and evaluating the impact of the results on patient outcomes. Answering if patient outcomes improved with increased teamwork and collaboration of the interprofessional team. This would require a larger sample size of trainees involved in simulation training.

### Conclusions

We were challenged with the reality of executing a research protocol in a highly complex health care environment, for example, clinician availability, time, response, ability for follow-up, change in protocol, data collection from clinical staff, etc. While these difficulties altered our study approach, the study team believes the design attempted in this study had merit in understanding the impact of a teamwork-focused ECMO simulation approach. We would encourage the medical community to build on the strengths of the design, fortify the weaknesses, and continue to emphasize the need for simulation training to improve ECMO care delivery and teamwork in the clinical setting. Especially as the field of simulation training continues to expand into new mediums like virtual reality [18].

## Supplementary material

10.2196/57424 Multimedia Appendix 1 Training curriculum.

10.2196/57424 Multimedia Appendix 2 Extracorporeal membrane oxygenation circuit.

10.2196/57424 Multimedia Appendix 3 Study timeline.

10.2196/57424 Multimedia Appendix 4 Qualitative interview guide.

10.2196/57424 Multimedia Appendix 5 Quantitative survey tool.

10.2196/57424 Multimedia Appendix 6 Amended study timeline.

10.2196/57424 Multimedia Appendix 7 Qualitative focus group themes.

10.2196/57424 Multimedia Appendix 8 Quantitative survey results.

## Abbreviations

CVI: Cardiac and Vascular Institute
ECMO: extracorporeal membrane oxygenation
HIPAA: Health Insurance Portability and Accountability Act
ICU: intensive care unit
USC: University of California
