# Supplementary material for: Barriers to and Facilitators of Implementing Team-Based Extracorporeal Membrane Oxygenation Simulation Study: Exploratory Analysis
Source: JMIR Med Educ. 2025 Jan 24;11:e57424. doi: 10.2196/57424 (PMC11788224; doi:10.2196/57424)
Supplement: Multimedia Appendix 1 [file mededu-v11-e57424-s001.docx]

**CURRICULUM FOR LOW-FIDELITY TEAM TRAINING**

| **COURSE TITLE** | Enhance Communication and Teamwork with Team Training |
| --- | --- |
| **TARGET AUDIENCE** | Clinical team involved in delivering ECMO therapy |
| **COURSE DESCRIPTION** | Interprofessional collaboration, specifically teamwork and communication, will be enhanced through a team training simulation using concepts from TeamSTEPPS®. Team roles and responsibilities, effective team-centered communication, and promoting patient-and-family centered care will be emphasized for patient care rounding. |
| **OBJECTIVES** | - Identifies two indications for VA ECMO - Identifies two major contraindications for VA ECMO (absolute or relative) - Identifies mechanism for activating ECMO at keck - Participates in Peripheral VA ECMO Initiation   - Identifies 4 team members and their roles in ECMO initiation   - Verbalizes considerations for cannulation   - Identifies common sites for central vs peripheral cannulation   - Identifies medications and material needed for initiation - Verbalizes immediate complications of ECMO cannulation - Identifies components of the ECMO circuit - Identifies Hemodynamic impacts of initiation of ECMO - Long Term complications of ECMO - Discusses considerations for consenting for ECMO |
| **COURSE OUTLINE** | Team simulation training will be used to enhance interprofessional collaboration, teamwork and communication among the burn team members to promote patient-centered goal setting during interdisciplinary patient care rounds.  Team Structure:   - Clearly define the roles and responsibilities of each member of the ECMO team   Communication   - Demonstrate the ability to provide brief, clear, and specific information to the team - Use team-centered communication strategies to state the specific actions that team will take   Mutual Support   - Promote input from all members of the team - Demonstrate the ability to speak up and clarify information - Use closed loop communication between team members   Situation Monitoring   - Formulate at least one patient-centered goal for the plan of care |
| **METHODOLOGY and EVALUATION** | The two elements that follow presents a) the curriculum matrix for this presentation, which includes the teaching strategies and evaluation and b) the performance assessment. |
| **Patient Scenario** | 60-year-old female with PMHx of CAD, CKD, PVD. Admitted today with recurrent VT resistant to chemical and electrical cardioversion. Patient experiences intermittent runs of VT on monitor and is cardioverted multiple times, ultimately experiencing persistent VT resistant to cardioversion or medication  Team decision making should lead to a decision to evaluate for placement of peripherally cannulated VA ECMO. Simulation requires ongoing code response and team members to attempt cannulation.  Once cannulated, patient stabilizes |

| **Title: Team Simulation Training to Enhance Communication and Teamwork** | | | | |
| --- | --- | --- | --- | --- |
| **Student Learning Outcomes/Objectives** | **Content/Outline** | **Teaching Strategies** | | **Evaluation** |
|  |  | **Faculty Activity** | **Student Activity** |  |
| 1. Discriminate the roles and responsibilities of each member of the burn team 2. Demonstrate the ability to provided brief, clear, and specific information to the team 3. Use team-centered communication strategies to state the action the team will take 4. Promote input from all members of the team 5. Demonstrate the ability to speak up and clarify information 6. Formulate at least one patient-centered goal for the plan of care | **Team Structure:** Burn Team Roles and Responsibilities document (see Appendix A)  **Communication:**   - Use the interdisciplinary plan of care document for the team to follow the patient care rounding elements (see Appendix B) - Shared responsibility and states the action the team will take using “we will do” or “let’s” phases (team centered vs. status differences)   **Mutual Support:**   - Essential elements of feedback (see Appendix C) - CUS tool for advocacy, assertion and mutual support (see Appendix D)   **Situation Monitoring:**  The section of “Top goals and Priorities” on the interdisciplinary plan of care will be used for patient-centered goals for the week | 1. The facilitator will use the document “Burn Team Roles and Responsibilities” to review team roles and responsibilities with burn team members. 2. The facilitator will explain and demonstrate the components of the interdisciplinary plan of care and expected flow of discussion during the patient care rounds 3. The facilitator will present components of team-centered communication using statement that distinguish team-centered communication versus communication that promotes status differences among team members 4. The facilitator will present the essential elements of feedback 5. The facilitator will present the CUS tool for advocacy, assertion and mutual support 6. The facilitator will provide examples of patient-centered weekly goals | 1. The learner will provide input to the plan of care based on their scope of practice and the “Burn Team Roles and Responsibilities” document.   1. The learner will follow the prompts of the interdisciplinary plan of care during the simulation exercise 2. The learner will use “we” and “let’s”, rather the “I”, “you” 3. The learner will give feedback during simulation exercise using the essential elements for providing feedback (see Appendix C) 4. The learner will resolve a safety issue during the simulation exercise using CUS (see Appendix D) 5. The learner will formulate a patient-centered goal during the simulation exercise | 1. The team score on the observation form on the component of Team Structure will be a score of Average or above 2. The team score on the observation form on the component of Communication will be a score of Average or above 3. The team score on the observation form on the component of Mutual Support will be a score of Average or above 4. The team score on the observation form on the component of Situation Monitoring will be a score of Average or above |

**Performance Assessment of Interprofessional Collaboration – Teamwork and Communication**

Observer Form

| Names of Participant:  1. 5.  2. 6.  3. 7.  4. 8. | Role:  1. 5.  2. 6.  3. 7.  4. 8. | | | | | | |
| --- | --- | --- | --- | --- | --- | --- | --- |
| Scenario: ECPR for recurrent VTach, initiation of Peripheral VA ECMO | | | | | | | |
| Observer: *From your perspective as a team observer, how would you describe the performance of this team? You will not describe individual performance but will focus on describing team functioning.* | | | | | | | |
| Skill Domains | | Poor |  | Average |  | Excellent | Comments |
| **Team Structure** | |  |  |  |  |  |  |
| - Clearly defines the roles and responsibilities of each member of the ECMO team - Assesses Leadership performance | |  | | | | |  |
| **Communication** | |  |  |  |  |  |  |
| - Demonstrate the ability to provide brief, clear, and specific information to the team - Use team-centered communication strategies to state the specific actions that team will take - Identifies need for ECMO Cannulation collaboratively | |  | | | | |  |
| **Mutual Support** | |  |  |  |  |  |  |
| - Promote input from all members of the team - Demonstrate the ability to speak up and clarify information - Uses closed loop communication with actions performed in the simulation. - Utilized the 2 Challenge rule when appropriate | |  | | | | |  |
| **Situational Monitoring** | |  |  |  |  |  |  |
| - Formulate at least one patient-centered goal for the plan of care | |  | | | | |  |

**Poor:** Multiple behaviors absent or not performed well; **Average:** Most behaviors preset and adequately performed; **Excellent:** All behaviors present and performed well.
