# Supplementary material for: Barriers to and Facilitators of Implementing Team-Based Extracorporeal Membrane Oxygenation Simulation Study: Exploratory Analysis
Source: JMIR Med Educ. 2025 Jan 24;11:e57424. doi: 10.2196/57424 (PMC11788224; doi:10.2196/57424)
Supplement: Multimedia Appendix 2 [file mededu-v11-e57424-s002.docx]

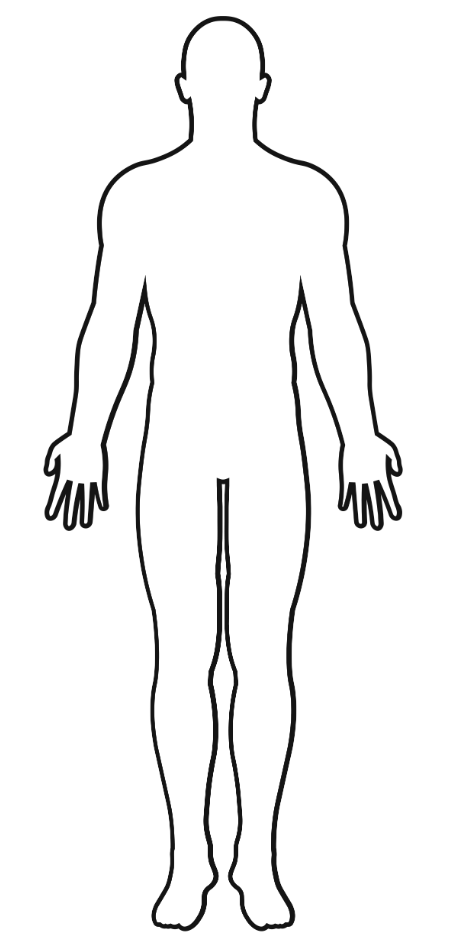

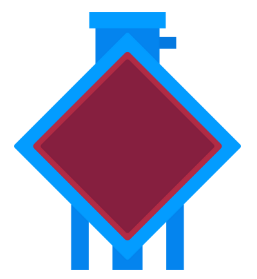
SDC Figure 1: Extracorporeal Membrane Oxygenation: VA-ECMO

Areas of Safety Concerns with ECMO Support

1. Vascular access: Vascular access is performed percutaneously at the bedside. The femoral vein is accessed for blood withdraw and the femoral artery is accessed for blood return. There is a significant risk of bleeding, vessel perforation, decreased distal limb perfusion. Relative contraindication including peripheral vascular disease may affect the choice of cannulation site or the ability of the patient to receive ECMO. Large volumes of blood flow and pressure differentials at the access site can rapidly result in air entrainment into the circuit, air embolism, or hemorrhage.
2. Centrifugal blood pump: Motor failure can result in abrupt cessation of care. A back-up pump is required for the duration of therapy. Adjustment of motor speed directly affects the level of perfusion delivered and is sensitive to patient volume status. Initiation of support is closely controlled by the interdisciplinary team.
3. Oxygenator function: Oxygen diffuses across the membrane into the blood and CO2 diffuses out of the blood. Amount of diffusion is controlled by an external gas blender and must be titrated to arterial blood gas results to prevent rapid changes in blood chemistry that may precipitate complications. Additionally, the membrane can accumulate thrombus and eventually fail, requiring replacement.

*c.*

*b.*

*a.*

Oxygenated blood

Deoxygenated blood

Centrifugal Blood Pump

Oxygenator
