# Supplementary material for: Barriers to and Facilitators of Implementing Team-Based Extracorporeal Membrane Oxygenation Simulation Study: Exploratory Analysis
Source: JMIR Med Educ. 2025 Jan 24;11:e57424. doi: 10.2196/57424 (PMC11788224; doi:10.2196/57424)
Supplement: Multimedia Appendix 3 [file mededu-v11-e57424-s003.pdf]

Qualitative Interview Timeline

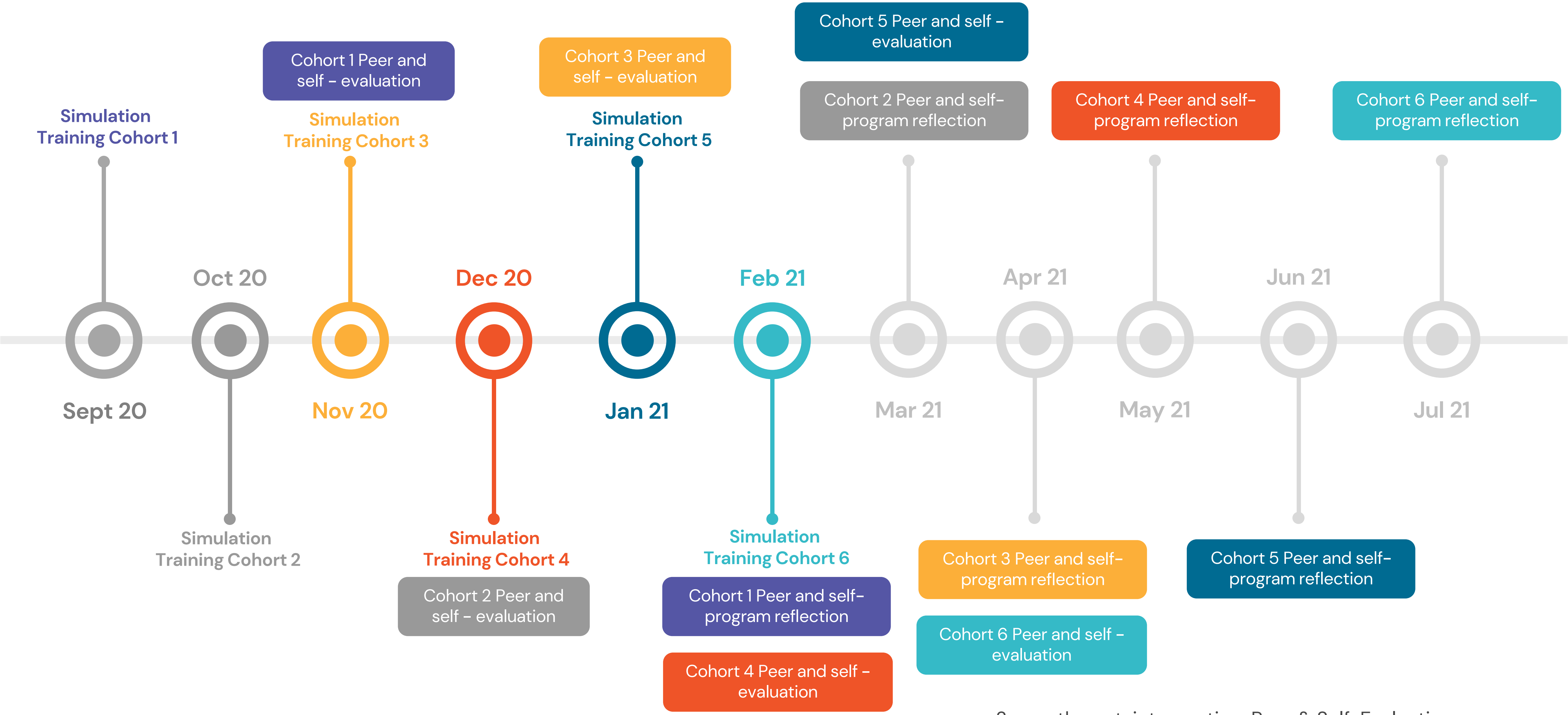

- 2-month post-intervention: Peer & Self-Evaluation
- 5-month post-intervention : Peer & Self- Program Reflection
