# Supplementary material for: Barriers to and Facilitators of Implementing Team-Based Extracorporeal Membrane Oxygenation Simulation Study: Exploratory Analysis
Source: JMIR Med Educ. 2025 Jan 24;11:e57424. doi: 10.2196/57424 (PMC11788224; doi:10.2196/57424)
Supplement: Multimedia Appendix 4 [file mededu-v11-e57424-s004.docx]

**SDC Table 3.** Qualitative Interview Protocol

Detailed interview process followed for trainee and peer focus groups at all intervals

**Peer Evaluation Focus Group**

Setup

- Thank you for taking the time to participate in this peer evaluation.
- This session will last approximately 1 hour
- We will be recording the video and audio for documentation purposes via Microsoft teams
- We may interrupt you to be mindful of your time and to get through all the questions.
- Keep in mind, there are no right or wrong answers; our aim is to understand your perspective

Purpose

- The purpose of the focus group is to evaluate the impact of the ECMO simulation training on (insert names of trainee cohort) completed on (insert date)
- Do you have any questions before we begin? Great, let’s get started.

Questions

- This group [name the individuals] went through training on X date focused on enhancing communication and teamwork during ECMO simulation. Can you comment on any improvements you’ve observed in their clinical judgment since the training?
  - Probe: How did these individuals work with the clinical team before the training?
- How easy is it for them to facilitate consensus toward cannulating a patient?
  - What steps have they taken to reach a team decision?
  - Where have they been getting stuck?
- How has their ability to resolve disagreements across the interprofessional team changed?
  - Probe: can you share some examples of how disagreements you observed and how they navigated them?
- Can you give me some examples on how the trainee cohort has demonstrated a clear sense of roles and responsibilities?
- What has been the individuals process to formulate a patient-centered goal for the plan of care?
  - Probe: Which steps are they having difficulty with?
- Talk me through how they promote input from all team members.
  - Probe: How might they improve in this area?
- What other opportunities for improvement does this trainee cohort have as it relates to their ability to facilitate ECMO therapy and foster a team environment?
- Any additional comments on the impact of the simulation training on these individuals?

**Trainee Evaluation Focus Group**

Setup

- Thank you for taking the time to participate in this training evaluation.
- This session will last approximately 1 hour
- We will be recording the video and audio for documentation purposes via Microsoft teams
- We may interrupt you to be mindful of your time and to get through all the questions.
- Keep in mind, there are no right or wrong answers; our aim is to understand your perspective

Purpose

- The purpose of the focus group is to evaluate the impact of the ECMO simulation training on your daily practice
- Do you have any questions before we begin? Great, let’s get started.

Questions

- Prior to the simulation training, can you describe any difficulty you had in leading the clinical team to decide on ECMO therapy?
- How has the simulation training impacted your ability to lead the clinical team?
- What skills do you feel you’ve gained because of the training?
  - Probe: Can you share examples of how you’ve applied these skills?
  - What has remained a challenge for you?
- What are the steps you take to resolve disagreements when trying to decide to cannulate a patient?
- What are the roles and responsibilities of each of the clinical team members when evaluating a patient for ECMO therapy?
- Can you talk me through how you formulate the plan of care for the ECMO patient candidates?
  - Probe: Are any of these steps more challenging than others? What might make them less difficult?
- Any additional comments on the impact of the simulation training on your daily practice?

**Peer Program Reflection**

Setup

- Thank you for taking the time to participate in this program evaluation.
- Like the first focus group, this session will last approximately 1 hour
- We will be recording the video and audio for documentation purposes via Microsoft teams
- We may interrupt you to be mindful of your time and to get through all the questions.
- Keep in mind, there are no right or wrong answers; our aim is to understand your perspective

Purpose

- Like the focus group in month 2, the purpose of this session is to evaluate the impact of the ECMO simulation training on (insert names of trainee cohort) completed on (insert date)
- Do you have any questions before we begin? Great, let’s get started.

Questions

- This group [name the individuals] went through training on X date focused on enhancing communication and teamwork during ECMO simulation.
- What impact, if any, have you observed in this trainee cohort since the simulation training?
  - Probe: Can you give any examples of how they’ve sustained this behavior in clinical practice?
- What areas of improvement remain within this cohort?
- How has participation in this training changed the culture on the unit if at all?
- Are there any other role groups you would include in this training?
- What areas of improvement still need to be addressed in this trainee cohort as it relates to teamwork?

**Trainee Program Reflection**

Setup

- Thank you for taking the time to participate in this program evaluation.
- Like the first focus group, this session will last approximately 1 hour
- We will be recording the video and audio for documentation purposes via Microsoft teams
- We may interrupt you to be mindful of your time and to get through all the questions.
- Keep in mind, there are no right or wrong answers; our aim is to understand your perspective

Purpose

- Like the focus group in month 2, the purpose of this session is to evaluate the impact of the ECMO simulation training on your daily practice
- Do you have any questions before we begin? Great, let’s get started.

Questions

- How has the simulation training changed your ability to coordinate care across the clinical team?
- How difficult is it to gain consensus on the plan of care?
  - Probe: In what ways has this changed for you when comparing your ability before and after the simulation training?
- What steps do you take to resolve conflict among the clinical team?
  - Probe: Which steps are more challenging than others? Why?
- If you could redesign the simulation training in any way, what would that look like?
