# Supplementary material for: Barriers to and Facilitators of Implementing Team-Based Extracorporeal Membrane Oxygenation Simulation Study: Exploratory Analysis
Source: JMIR Med Educ. 2025 Jan 24;11:e57424. doi: 10.2196/57424 (PMC11788224; doi:10.2196/57424)
Supplement: Multimedia Appendix 5 [file mededu-v11-e57424-s005.docx]

**SDC Table 1.**

Quantitative Survey Tool

3 surveys were designed to engage trainees prior to simulation training (pre-), immediately following (post-) and 3-months post simulation to evaluate impact. The table lists each survey question and when it was used. The table also delineates the questions across the Kirkpatrick Training Evaluation Framework used to assess the training on four levels.

| Question | Pre | Post | 3-months |
| --- | --- | --- | --- |
| Knowledge Questions Y/N, and free text to indicate the 2 | | | |
| I am able to identify 2 Indications for VA ECMO (Y/N) | X | X |  |
| I am able to identify 2 major absolute or relative contraindication for VA ECMO (Y/N) | X | X |  |
| I am able to identify 2 immediate complications or peripherally cannulated VA ECMO (Y/N) | X | X |  |
| Level 1 Questions 5- point Likert Scale (strongly disagree, somewhat disagree, neither agree nor disagree, somewhat agree, strongly agree) | | | |
| I found this to be an effective use of my time. |  | X |  |
| I found the content was relevant to my job. |  | X |  |
| My learning was enhanced by this training. |  | X |  |
| This training improved my ability to deliver ECMO care. |  | X |  |
| I would recommend attending this training session to a colleague. |  | X |  |
| I am satisfied with the simulation training experience. |  |  | X |
| This training improved my ability to deliver ECMO care. |  |  | X |
| Level 2 Questions 5- point Likert Scale (strongly disagree, somewhat disagree, neither agree nor disagree, somewhat agree, strongly agree) | | | |
| I feel I gained essential teamwork skills needed to deliver ECMO care. |  | X |  |
| I feel comfortable using the ECMO equipment specific to my role | X | X | X |
| I feel comfortable using the 2-challenge rule |  | X | X |
| I understand the mechanism for activating ECMO at Keck Hospital | X | X | X |
| I understand my role in a bedside cannulation | X | X | X |
| I feel confident to voice concerns to leadership during a critical situation | X | X | X |
| The initiating team communicates efficiently during a bedside cannulation |  | X | X |
| I feel confident in my ability to deliver ECMO care |  | X | X |
| I understand my role and responsibilities to deliver ECMO care |  | X | X |
| I trust my colleagues to perform the processes of care needed to deliver ECMO care | X | X | X |
| Mayo High Performance Teamwork Scale: 0 Never or rarely, 1 Inconsistently, 2 Consistently | | | |
| A leader is clearly recognized by all team members | X | X |  |
| The team leader assures maintenance of an appropriate balance between command authority and team member participation | X | X |  |
| Each team member demonstrates a clear understanding of his or her role | X | X |  |
| The team prompts each other to attend to all significant clinical indicators throughout the procedure/intervention | X | X |  |
| When team members are actively involved with the patient, they verbalize their activities aloud | X | X |  |
| Team members repeat back or paraphrase instructions and clarifications to indicate that they heard them correctly | X | X |  |
| Team members refer to established protocols and checklists for the procedure/intervention | X | X |  |
| All members of the team are appropriately involved and participate in the activity | X | X |  |
| Mayo High Performance Teamwork Scale: 0 Never or rarely, 1 Inconsistently, 2 Consistently, NA | | | |
| Disagreements or conflicts among team members are addressed without a loss of situation awareness | X | X |  |
| When appropriate, roles are shifted to address urgent or emergent events | X | X |  |
| When directions are unclear, team members acknowledge their lack of understanding and ask for repetition and clarification | X | X |  |
| Team members acknowledge-in a positive manner-statements directed at avoiding or containing errors or seeking clarification | X | X |  |
| Team members call attention to actions that they feel could cause errors or complications | X | X |  |
| Team members respond to potential errors or complications with procedures that avoid the error or complication | X | X |  |
| When statements directed at avoiding or containing errors or complications do not elicit a response to avoid or contain the error, team members persist in seeking a response | X | X |  |
| Team members ask each other for assistance prior to or during periods of task overload. | X | X |  |
| Level 3 Questions 5- point Likert Scale (strongly disagree, somewhat disagree, neither agree nor disagree, somewhat agree, strongly agree) | | | |
| I am using what I learned from the training in my daily work |  |  | X |
| Care coordination happens easily |  |  | X |
| There are no breakdowns in communication between team members |  |  | X |
| Feedback is given constructively |  |  | X |
| The feedback I receive motivates me to perform better |  |  | X |
| The organization's culture supports the collaboration required to deliver care |  |  | X |
| Interprofessional collaboration increased with the training |  |  | X |
| Level 4 Questions 5- point Likert Scale (strongly disagree, somewhat disagree, neither agree nor disagree, somewhat agree, strongly agree) | | | |
| The training helped our ICU reach our goals |  |  | X |
| The team has performed better since attending the training |  |  | X |
| Staff satisfaction has improved since the training |  |  | X |
| I feel the simulation training has improved ECMO patient outcomes |  |  | X |
| Since this training I was able to use the principles learned in other patient care activities |  |  | X |
