# Supplementary material for: Barriers to and Facilitators of Implementing Team-Based Extracorporeal Membrane Oxygenation Simulation Study: Exploratory Analysis
Source: JMIR Med Educ. 2025 Jan 24;11:e57424. doi: 10.2196/57424 (PMC11788224; doi:10.2196/57424)
Supplement: Multimedia Appendix 7 [file mededu-v11-e57424-s007.docx]

**SDC Table 5.** Qualitative Results

Theme, sub-themes, and the supporting direct quotes from the qualitative focus groups.

| **Theme** | **Direct Quote** |
| --- | --- |
| Working together as a stronger and more confident team because of simulation | “We had Pulm Crit [Pulmonary Critical Care Medicine] here, but also having [NAME 1] from the anesthesia side like us working together because that's literally how it is in the actual scenarios, versus just has me and [NAME 2] in the sim I don't think we would have known to call for blood and give heparin....” (Trainee -Speaker 3, 2022.07.29)  “The more exposure the better...at first it's kind of silly just to SIM, but then it really makes you look like, OK, this is what it's for and just working as a team. It's a great idea—makes everyone more comfortable for this situation we know is going to happen again.” (Trainee-Speaker 4, 2022.07.29) |
|  | Being able to come together to have shared goal, having space to resolve, checking in and making informed decision together; allow for team problem-solving  “[T]his patient needs to go on ECMO and [NAME] doesn't feel like it. For me to say ‘I think this’ and then for him to at least ask, ‘Why do you think so?’ And I'm like XY and Z and he can and instead of being like, ‘No!’ he can be like, ‘My concerns are because of this and this. Has anyone spoken to cardiology? Is this patient even a transplant candidate? Do we have an end goal?’ If we're not sure, we're going to do it and figure it out. It's allowing everyone to say their piece, but then also [voicing] this is my concern (Trainee-Speaker 3, 2022.07.29) |
|  | "Now just knowing that when we do call for it, if I'm the code lead, make it a point to go talk to the ECMO team lead and be communicating together versus like two different teams...[J]just communicating with the team and being like, ‘What do you need? What can we do? These are my concerns.’ ” (Trainee-Speaker 3, 2022.07.29) |
| Creating a space to improve communications, decision-making, and express concerns via simulation | “[ECMO simulation] helped me see things from a lot of different perspectives...it's good to also consider other people's opinions and...know other people's limitations...strengths and weaknesses.” (Trainee-Speaker 2, 2022.07.29)  “In terms of the cannulation strategy, sometimes you may actually want to hold off on the compression, so that it makes the surgeons job a little bit easier...You have to be paying attention to it, because otherwise the surgeon will...tell you [to] hold compressions and you will be like, ‘No, I want compressions.’ That's where you have to pay attention to what's going on and having that mindset [that] you need to anticipate what they're doing.” (Trainee-Speaker 2, 2022.08.26) |
|  | “Because there's going to be differences of opinions, just being calm in a high stress environment. The patient had malignancy three years ago and [it was] cool trying to involve the primary team.” (Trainee-Speaker 4, 2022.07.29) |
|  | “[Trainee] did not necessarily have to reconcile any disagreements because...he was the primary team and they were driving the ship. And he did recognize some of the concerns that were voiced, namely the concerns that I had indicated to him by telling him that, ‘This is not something that I would typically do’--namely meaning excavate a patient later in the daytime when I know that there is going to be lesser support available. However, his response was making sure that he did have a backup plan in mind should things not go smoothly.” (Peer-Speaker 2, 2022.03.31) “[The trainee] had the insight to know that that was potentially an issue” |
|  | “...[I]t's kind of chaotic, but I think that's kind of my big take away besides knowing when to call for it [call code ECMO]. But also then just communicating with the team and being like, ‘What do you need? What can we do? These are my concerns.’ ” (Trainee-Speaker 3, 2022.07.29) |
