# Supplementary material for: Barriers to and Facilitators of Implementing Team-Based Extracorporeal Membrane Oxygenation Simulation Study: Exploratory Analysis
Source: JMIR Med Educ. 2025 Jan 24;11:e57424. doi: 10.2196/57424 (PMC11788224; doi:10.2196/57424)
Supplement: Multimedia Appendix 8 [file mededu-v11-e57424-s008.docx]

**SDC Table 4.** Quantitative Survey Results

Survey participants responses to the pre- and post- survey

| Question | Pre/Post | Item Response Rate | Strongly Agree | Agree | Neither agree nor disagree | | Disagree | | Strongly Disagree | Agree Level | Disagree Level |
| --- | --- | --- | --- | --- | --- | --- | --- | --- | --- | --- | --- |
| I understand the mechanism for activating ECMO at Keck Hospital (level 2) | Pre | 100% | 7% | 39% | 18% | | 18% | | 18% | 46% | 36% |
|  | Post | 100% | 62% | 38% | 0% | | 0% | | 0% | 100% | 0% |
| I understand my role in a bedside cannulation (level 2) | Pre | 96% | 4% | 19% | 48% | | 11% | | 19% | 23% | 30% |
|  | Post | 100% | 46% | 54% | 0% | | 0% | | 0% | 100% | 0% |
| I feel comfortable using the ECMO equipment specific to my role (level 2) | Pre | 100% | 0% | 25% | 29% | | 32% | | 14% | 25% | 46% |
|  | Post | 100% | 31% | 58% | 12% | | 0% | | 0% | 89% | 0% |
| I feel comfortable using the 2- challenge rule (level 2) | Pre | 100% | 4% | 0% | 29% | | 25% | | 43% | 4% | 68% |
|  | Post | 100% | 38% | 31% | 15% | | 0% | | 15% | 69% | 15% |
| I feel confident to voice concerns to leadership during a critical situation (level 2) | Pre | 100% | 32% | 46% | 11% | | 11% | | 0% | 78% | 11% |
|  | Post | 100% | 69% | 27% | 4% | | 0% | | 0% | 96% | 0% |
| I found this to be an effective use of my time (level 1) | Post | 100% | 81% | 19% | 0% | | 0% | | 0% | 100% | 0% |
| I found the content was relevant to my job (level 1) | Post | 100% | 85% | 15% | 0% | | 0% | | 0% | 100% | 0% |
| My learning was enhanced by this training (level 1) | Post | 100% | 73% | 23% | 4% | | 0% | | 0% | 96% | 0% |
| This training improved my ability to deliver ECMO care (level 1) | Post | 100% | 65% | 31% | 4% | | 0% | | 0% | 96% | 0% |
| I would recommend attending this training session to a colleague (level 1) | Post | 100% | 69% | 27% | 4% | | 0% | | 0% | 96% | 0% |
| I feel I gained essential teamwork skills needed to deliver ECMO care (level 2) | Post | 100% | 65% | 31% | 4% | | 0% | | 0% | 96% | 0% |
| I feel confident in my ability to deliver ECMO care (level 2) | Post | 100% | 35% | 50% | 15% | | 0% | | 0% | 85% | 0% |
| I understand my role and responsibilities to deliver ECMO care (level 2) | Post | 100% | 54% | 38% | 8% | | 0% | | 0% | 92% | 0% |
| I trust my colleagues to perform the processes of care needed to deliver ECMO care (level 2) | Post | 100% | 65% | 27% | 0% | | 0% | | 0% | 92% | 0% |
| Question | Pre/Post | Item Response Rate | %Yes | %No | |  | |  |  |  |  |
| I am able to identify 2 indications for VA ECMO | Pre | 100% | 79% | 21% | |  | |  |  |  |  |
|  | Post | 100% | 92% | 8% | |  | |  |  |  |  |
| I am able to identify 2 major absolute or relative contraindications for VA ECMO (level 2) | Pre | 96% | 59% | 41% | |  | |  |  |  |  |
|  | Post | 87% | 87% | 13% | |  | |  |  |  |  |
| I am able to identify 2 immediate complications of peripherally cannulated VA ECMO (level 2) | Pre | 100% | 57% | 43% | |  | |  |  |  |  |
|  | Post | 92% | 88% | 13% | |  | |  |  |  |  |
| Question (teamwork scale) | Pre/Post | Item Response Rate | % Never or rarely | % Inconsistently | | % Consistently | |  |  |  |  |
| A leader is clearly recognized by all team members | Pre | 96% | 0% | 41% | | 59% | |  |  |  |  |
|  | Post | 87% | 0% | 17% | | 83% | |  |  |  |  |
| The team leader assures maintenance of an appropriate balance between command authority and team member participation | Pre | 96% | 0% | 19% | | 81% | |  |  |  |  |
|  | Post | 87% | 0% | 4% | | 96% | |  |  |  |  |
| Each team member demonstrates a clear understanding of his or her role | Pre | 96% | 0% | 37% | | 63% | |  |  |  |  |
|  | Post | 92% | 0% | 8% | | 92% | |  |  |  |  |
| The team prompts each other to attend to all significant clinical indicators throughout the procedure/intervention | Pre | 96% | 0% | 26% | | 74% | |  |  |  |  |
|  | Post | 92% | 0% | 8% | | 92% | |  |  |  |  |
| When team members are actively involved with the patient, they verbalize their activities aloud | Pre | 96% | 0% | 30% | | 70% | |  |  |  |  |
|  | Post | 92% | 0% | 8% | | 92% | |  |  |  |  |
| Team members repeat back or paraphrase instructions and clarifications to indicate that they heard them correctly | Pre | 96% | 0% | 37% | | 63% | |  |  |  |  |
|  | Post | 92% | 0% | 8% | | 92% | |  |  |  |  |
| Team members refer to established protocols and checklists for the procedure/intervention | Pre | 96% | 0% | 33% | | 67% | |  |  |  |  |
|  | Post | 92% | 0% | 13% | | 88% | |  |  |  |  |
| All members of the team are appropriately involved and participate in the activity | Pre | 96% | 0% | 22% | | 78% | |  |  |  |  |
|  | Post | 92% | 0% | 0% | | 100% | |  |  |  |  |
| Disagreements or conflicts among team members are addressed without a loss of situation awareness | Pre | 78% | 0% | 30% | | 70% | |  |  |  |  |
|  | Post | 87% | 0% | 4% | | 96% | |  |  |  |  |
| When appropriate, roles are shifted to address urgent or emergent events | Pre | 88% | 0% | 28% | | 72% | |  |  |  |  |
|  | Post | 92% | 0% | 4% | | 96% | |  |  |  |  |
| When directions are unclear, team members acknowledge their lack of understanding and ask for repetition and clarification | Pre | 83% | 0% | 25% | | 75% | |  |  |  |  |
|  | Post | 87% | 0% | 0% | | 100% | |  |  |  |  |
| Team members acknowledge-in a positive manner-statements directed at avoiding or containing errors or seeking clarification | Pre | 88% | 0% | 28% | | 72% | |  |  |  |  |
|  | Post | 92% | 0% | 4% | | 96% | |  |  |  |  |
| Team members call attention to actions that they feel could cause errors or complications | Pre | 88% | 0% | 28% | | 72% | |  |  |  |  |
|  | Post | 92% | 0% | 0% | | 100% | |  |  |  |  |
| Team members respond to potential errors or complications with procedures that avoid the error or complication | Pre | 88% | 0% | 24% | | 76% | |  |  |  |  |
|  | Post | 92% | 0% | 4% | | 96% | |  |  |  |  |
| When statements directed at avoiding or containing errors or complications do not elicit a response to avoid or contain the error, team members persist in seeking a response | Pre | 88% | 0% | 32% | | 68% | |  |  |  |  |
|  | Post | 87% | 0% | 0% | | 100% | |  |  |  |  |
| Team members ask each other for assistance prior to or during periods of task overload | Pre | 88% | 0% | 32% | | 68% | |  |  |  |  |
|  | Post | 92% | 0% | 0% | | 100% | |  |  |  |  |
